# A novel bi-alleleic *DDX41* mutations in B-cell lymphoblastic leukemia: case report

**DOI:** 10.1186/s12920-022-01191-2

**Published:** 2022-03-04

**Authors:** Woo Yong Shin, Seug Yun Yoon, Rojin Park, Jung-Ah Kim, Ho Hyun Song, Hae In Bang, Jong-Ho Won, Jieun Kim

**Affiliations:** 1 Department of Laboratory Medicine, Soonchunhyang University Seoul Hospital, Soonchunhyang University College of Medicine, Seoul, Korea; 2 Division of Hematology and Oncology, Department of Internal Medicine, Soonchunhyang University Seoul Hospital, Soonchunhyang University College of Medicine, Seoul, Korea; 3 Department of Interdisciplinary Program in Biomedical Science, Graduate School, Soonchunhyang University, Asan, Chungcheongnam-do Korea

**Keywords:** DDX41 germline mutation, B**-**cell lymphoblastic leukemia, Gene expression, Case report

## Abstract

**Background:**

The germline mutations of *DDX41*, also known as DEAD box RNA helicase 41, have been found in about 1.5% of myeloid neoplasms (MNs). Development of MDS/AML is relatively common in germline *DDX41* mutations. However, a variety of hematological malignancies (HMs) have been reported.

**Case presentation:**

We report a novel case of bi-alleleic DDX41 mutations in B-cell lymphoblastic leukemia (B-ALL), with unusual location of DDX41 mutations. The gene expression profile (GEP) of Ph + B-ALL with bi-alleleic DDX41 mutations showed heterogeneously transitional GEP and altered gene expression levels of genes involved in the process essential for red blood cells and myeloid cell differentiation were noted.

**Conclusions:**

We report that *DDX41* mutations are unusual but can be an underlying event in Ph + B-ALL and screening *DDX41* mutations can be also informative for patients awaiting for haploidentical stem cell transplantation and choosing the therapy.

**Supplementary Information:**

The online version contains supplementary material available at 10.1186/s12920-022-01191-2.

## Background

The awareness of the hereditary basis for hematologic malignancies (HMs) is increasing, such germline mutations are found in 4.4% to 18% of HM patients, depending on population [1]. The germline mutations of *DDX41*, also known as DEAD box RNA helicase 41, have been found in about 1.5% of myeloid neoplasms (MNs) [2]. Families with *DDX41* mutations display an autosomal dominant inheritance, with a clinical picture dominated by late onset of either myelodysplastic syndrome (MDS) or acute myeloid leukemia (AML) [3].

Development of MDS/AML is relatively common in germline *DDX41* mutations. However, a variety of hematological malignancies (HMs) have been reported, including rare cases of chronic myeloid leukemia (CML), and lymphoma, which means that mutations in *DDX41* cannot be attributed to a specific malignant disorder [4]. Therefore, an association between the types of *DDX41* mutations, accumulation of secondary mutations, or type of leukemia may pertain to its role in the leukemogenesis.

The processes by which the *DDX41* mutation contributes to the oncogenesis that leads to myeloid neoplasms (MNs) have been investigated, but the underlying molecular pathogenesis of *DDX41* mutations in B lymphoblastic leukemia (B-ALL) has not been revealed. Genetic expression profiling (GEP) has previously proven useful in B-ALL for identifying signatures of oncogenes, with the recognition of novel subgroups, as well as with outcome [5]. Therefore, we adopted GEP of a novel case of B-ALL with t(9;22) *BCR-ABL1* harboring *DDX41* germline and somatic mutations, to uncover the contribution of *DDX41* to leukemogenesis. In addition, we compared the GEP of the present case with other relevant samples, including B-ALL with t(9;22) *BCR-ABL1* and AML with bi-alleleic *DDX41* mutations, providing cluster analysis and thereby taking a step closer to understanding of the underlying mechanisms.

## Case presentation

### Case description

A 48-year-old man with a past medical history of hypertension, hyperlipidemia, and asthma presented with fatigue, and night sweats, and there were no palpable lymph nodes in his physical examination. Initial complete blood- cell counts included a hemoglobin (Hb) of 12.5 g/dL, white blood- cell (WBC) count of 22.1 × 10^9^/L with 67% blasts on peripheral blood smear, and a platelets count of 91 × 10^9^/L. In the bone- marrow (BM) aspiration, blasts accounted for 88.1% of ANCs, which were positive for CD9, CD10, CD13, CD19, CD20, CD34, CD38, CD58, CD66c, CD123, HLA-DR, cCD79a, and TdT on flow cytometry. The karyotype was revealed as 46,XY,t(9;22)(q34;q11.2)[10]/47,idem, + der(22)t(9;22)[2] by chromosomal tests. *BCR-ABL1* fusion was detected by FISH analysis and confirmed as a major transcript (b3a2). There was no specific familial history for any hematologic malignancies.

### Genomic sequencing and microarray analysis of expression profiles in samples

**Fig. 1:**
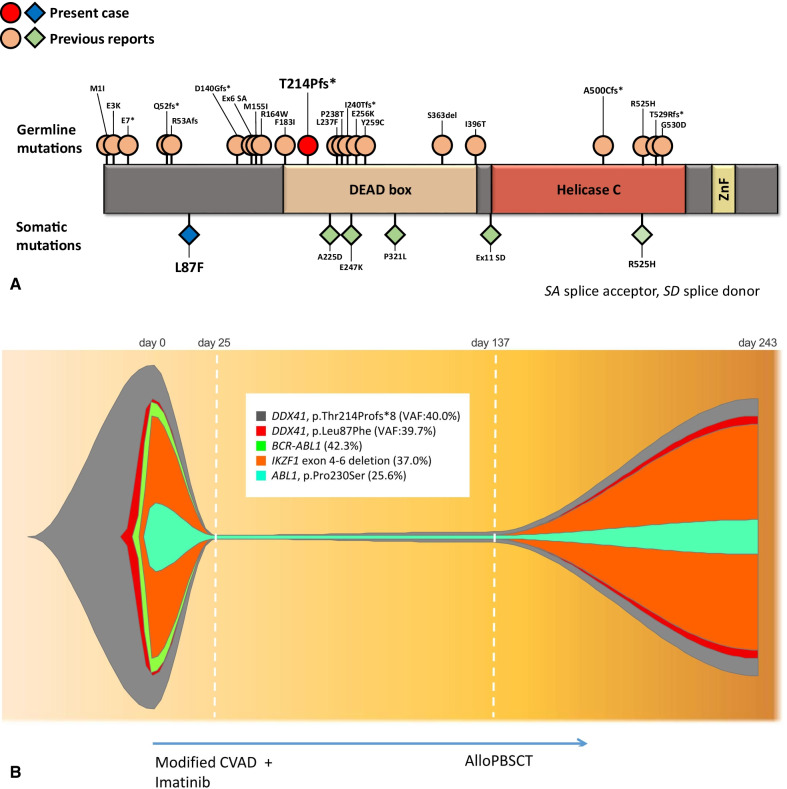
**A** Schematic representation of the reported germline and somatic *DDX41* mutations in hematologic malignancies and present study **B** Clonal architectures of our case series during the course of treatment. Variant allele frequency (VAF) for each mutation is indicated

*IKZF1* exon 4 ~ 6 somatic deletion was detected in the copy number variant analysis of the next- generation sequencing (NGS). Aside from the somatic *ABL1* mutation (c.688C > T, p.Pro230Ser), we found two novel *DDX41* mutations, c.639delC, p.Thr214Profs*8 and c.259C > T, p.Leu87Phe, and confirmed the frameshift variant of *DDX41* being of germline origin by Sanger sequencing of skin fibroblast (Fig. 1A, B) (see Additional file 4). Both mutations were not reported in gnomAD, 1000 Genomes, and HGVD database. Sorting Intolerant From Tolerant (SIFT) calculated that p.Leu87Phe has deleterious effect and PolyPhen-2 predicted to be probably damaging.

Genetic analysis was performed on five patients, including the present study (Ph + B-ALL^*IKZF1*+/*DDX41dm*^), two cases of Ph + B-ALL accompanying *IKZF1* deletion without *DDX41* mutation (Ph + B-ALL^*IKZF1*+/*DDX41−*^), one case of AML with double *DDX41* mutation (AML^*DDX41 dm*^), and one case of normal BM, whom were examined for HM at Soonchunhyang University Seoul Hospital, South Korea, from November 2018 to May 2020. This study was approved by the Institutional Review Board of Soonchunhyang University Seoul Hospital (IRB no. 2021–01-003). The fresh BM, whole blood (WB), and skin fibroblast specimens were stored at -80 °C before genome sequencing and GEP.

Briefly, genomic DNA were extracted from proband’s skin fibroblast, WB, and BM samples at initial diagnosis using a QIAamp DNA Blood Mini Kit (Qiagen, MD, USA) according to standard procedures. Targeted NGS with a hematologic malignancy comprehensive panel (Celemics, Seoul, South Korea), which examines 85 hematologic malignancy- associated genes (see Additional file 1) were performed in all samples. We confirmed sequence mutations and exonal deletions by Sanger sequencing and multiplex ligation-dependent probe amplification (MLPA) (SALSA MLPA P335-C1 ALL-IKZF1 probemix, MRC Holland, Amsterdam, Holland), respectively.

To determine whether two mutations in different regions of the *DDX41* of the patient were in different alleles, the cDNA including region of interest was amplified with the following primers (Forward, 5′-gaggaagagcagcaggacag-3′; Reverse, 5′-tcatgtcacggccagataga-3′). The PCR product was then cloned into the TA-cloning vector (Topcloner TA kit; Enzynomics, Daejeon, Korea) and ten clones were sequenced which included the corresponding regions of the *DDX41*.

RNA was extracted from proband’s BM samples at initial diagnosis using an RNeasy Micro kit (Qiagen) and then evaluated with an Agilent 2100 Bioanalyzer (Agilent Technologies, Santa Clara, USA) for RNA integrity. We obtained a total of 3.5 µg of cDNA after amplification with a GeneChip™ WT Pico Kit (Affymetrix, CA, USA) and processed it for GEP. GEPs were generated using the GeneChip Human Gene 2.0 ST Array (Affymetrix). A robust multi-average (RMA) method implemented in Affymetrix® Power Tools (APT) was used for data summarization and normalization. The results were exported to gene-level RMA analysis and differentially expressed gene (DEG) analysis was performed. Statistical significance of the expression data was determined using fold change. Gene-enrichment and functional annotation analysis for a significant probe list was performed using Gene Ontology (http://geneontology.org) and KEGG (Kyoto Encyclopedia of Genes and Genomes, http://kegg.jp). All data analysis and visualization of differentially expressed genes was conducted using R 3.3.2 (The R Foundation for Statistical Computing, Vienna, Austria).

We compared the differentially expressed genes of Ph + B-ALL^*IKZF1*+/*DDX41dm*^ with two cases of Ph + B-ALL^*IKZF1*+/*DDX41−*^ in order to elucidate the role of *DDX41* in leukemogenesis. Assessment of differential expression between samples was conducted employing linear models for microarrays in R, and genes with fold change ≥ 3 and *p* < 0.05 were to be considered significant. For a DEG set, hierarchical cluster analysis was done in all samples in order to assess the degree of relatedness; and samples were clustered using complete linkage and Euclidean distance as a measure of similarity.

## Results

The patient visited this medical center outpatient for 17 years before being diagnosed, and except for April 2015, when neutrophils were elevated due to pneumonia, WBC count was 6.1–10.0 × 10^9^/L and differential count was normal. The last visit was 3 years before diagnosis and there was no CML related morphologic evidence such as basophilia and myeloid proliferation at diagnosis. Therefore, it was determined that there would be no underlying disease such as CML, the patient was diagnosed as B-ALL with t(9;22)(q34.1;q11.2) and received induction chemotherapy of modified Hyper-CVAD regimen (cyclophosphamide, vincristine, adriamycin, dexamethasone and pegylated asparaginase) with imatinib. After a month of induction therapy, blasts were decreased to 0.1% of ANCs in the follow-up BM exam, and *BCR-ABL1* transcripts were decreased to < 0.0004% on the international scale. The patient received allogenic peripheral blood stem cell transplantation (SCT) from a sibling donor without germline *DDX41* mutation after consolidation chemotherapy with high-dose cytarabine and mitoxantrone. However, B-ALL relapsed nine months later, with an increased WBC count of 85.2 × 10^9^/L with 81% blasts on peripheral blood.

**Fig. 2:**
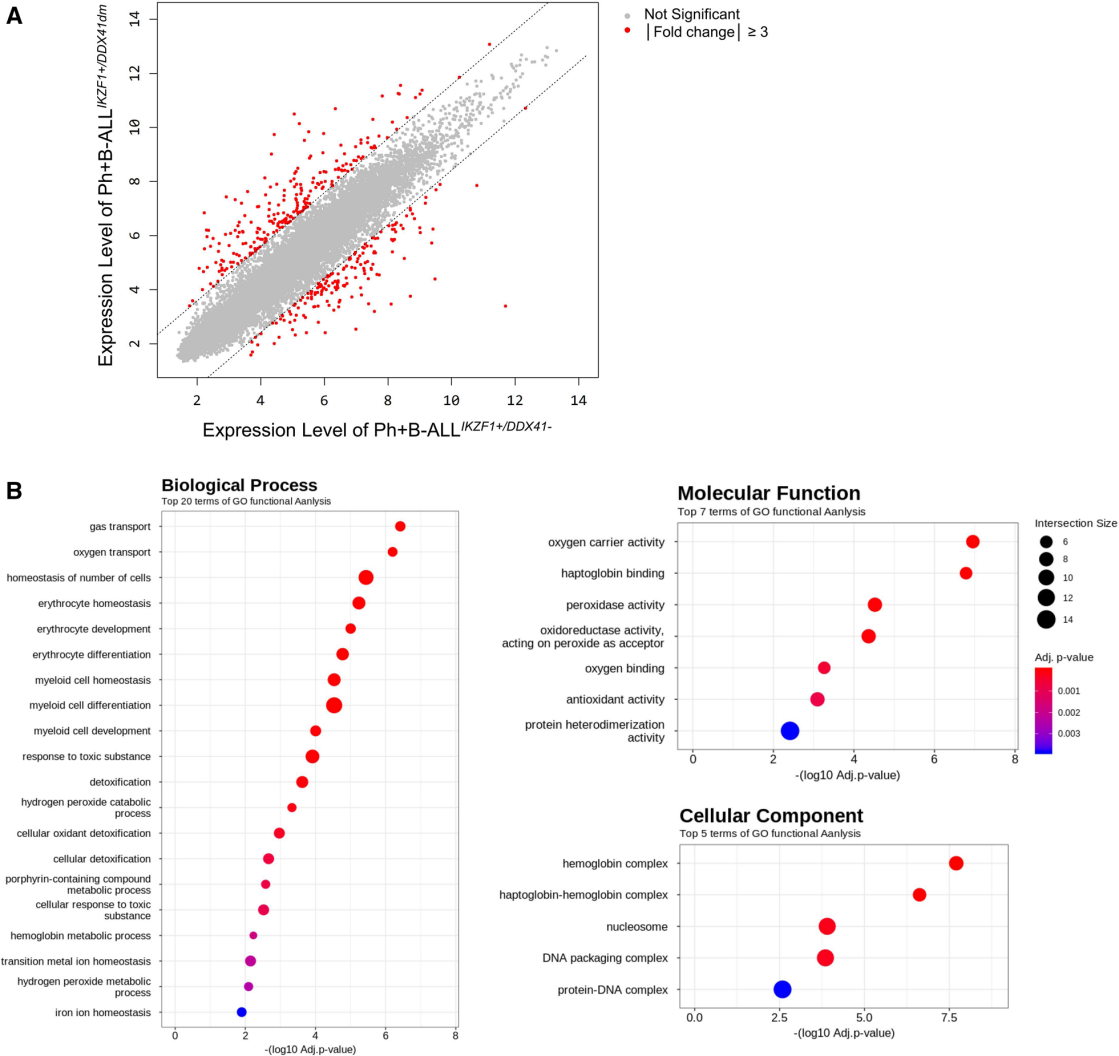
**A** Microarray analysis of differentially expressed genes in BM sample obtained from patient with Ph + B-ALL^*IKZF1*+/*DDX41dm*^. Up- and down-regulated genes are represented in red. **B** GO analysis of transcriptome in BM of patient with Ph + B-ALL^*IKZF1*+/*DDX41dm*^. The X-axis represents the − log10 (*P* value) of the given transcripts and the Y-axis shows a detailed description of the roles for the category

Clinical features and genetic alterations detected in genomic DNA sequencing of all samples are shown (see Additional file 2); and the clonal architecture of the present case during the course of treatment are is depicted in Fig. 1. Two mutations, c.639delC and c.259C > T, were confirmed to be present in different alleles. Of 53,617 transcripts represented by the microarray, 409 were differentially over- or under-expressed in a Ph + B-ALL^*IKZF1*+/*DDX41dm*^ sample as compared to Ph + B-ALL^*IKZF1*+/*DDX41−*^ samples. Among those 409 transcripts, 233 were expressed more abundantly and 176 less abundantly in Ph + B-ALL^*IKZF1*+/*DDX41dm*^ (Fig. 2A). A list of the 409 dysregulated transcripts for Ph + B-ALL^*IKZF1*+/*DDX41dm*^ is shown in more detail (see Additional file 3). To summarize, among DEGs, the expression of DDX41 in case sample (Ph + B-ALL^*IKZF1*+*/DDX41dm*^) was characterized by high-level expression of a set of genes involved in p53 signaling pathway when compared to Ph + B-ALL^*IKZF1*+*/DDX41−*^ samples, whereas B cell receptor signaling pathway, PI3K-Akt signaling pathway, and NF-kappa B signaling pathway were differentially expressed compared to AML ^*DDX41dm*^.

**Fig. 3:**
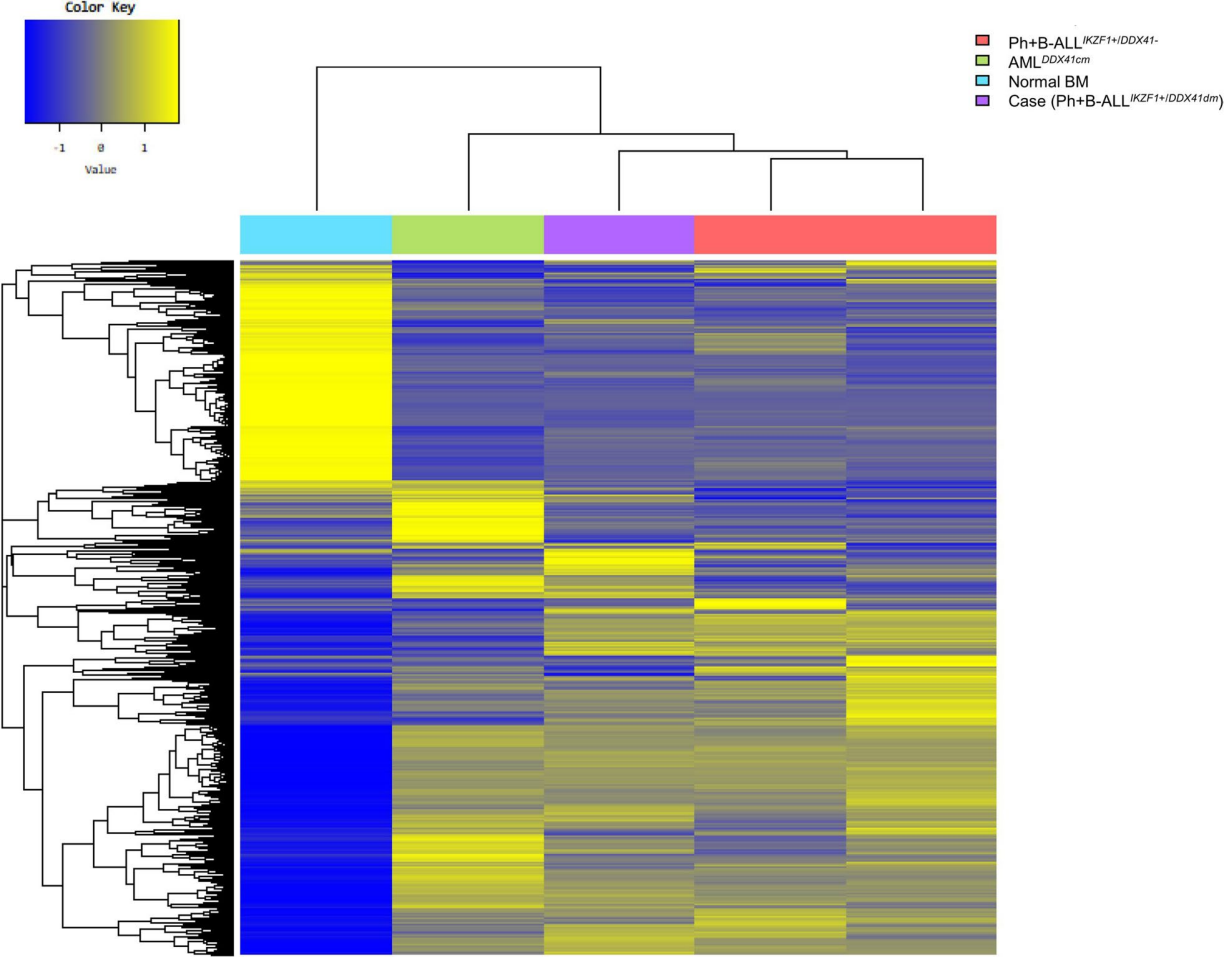
Hierarchical cluster analysis of five BM samples. The heatmap combines GEP of Ph + B-ALL^*IKZF1*+/*DDX41*^*,* Ph + B-ALL^*IKZF1*+/*DDX41−*^, AML^*DDX41dm*^ and normal BM samples

To elucidate the biological significance of differentially expressed genes in Ph + B-ALL^*IKZF1*+/*DDX41dm*^, gene ontology (GO) analysis of the whole transcriptome was performed. Of the 3,214 functional categories examined, the top DEGs are presented in Fig. 2B. GO analysis revealed that significant categories for expressed genes seem to have a strong correlation with the process essential for red blood cells and myeloid cells. Pathway enrichment revealed that there were 11 significant pathways were enriched in Ph + B-ALL^*IKZF1*+/*DDX41dm*^ compared to Ph + B-ALL^*IKZF1*+/*DDX41−*^, and one of the most significant pathways was transcriptional misregulation in cancer (hs05202) genes (*p* < 0.001), which contained common cancer-related genes, such as *HIST1H3G, SUPT3H, HIST1H3I, BCL2L1, WT1, HIST1H3A, CD86, CDK14, CSF1R,* and *PROM1.* Detailed analysis of the dysregulated genes revealed several candidates linked to relevant signaling pathways in Ph + B-ALL^*IKZF1*+/*DDX41dm*^, which may represent pathogenetically relevant genes. Genes associated with proliferation, and cell survival (*BCL2L1*), and tumor- cell growth (*WT1*) pathways for development of cancer were overexpressed, whereas molecules relevant to differentiation resistance (*CSF1R*) were underexpressed. Based on hierarchical cluster analysis, Ph + B-ALL^*IKZF1*+/*DDX41dm*^ can be distinguished from Ph + B-ALL^*IKZF1*+/*DDX41−*^ and AML ^*DDX41dm*^ and that the nature of Ph + B-ALL^*IKZF1*+/*DDX41dm*^ is closer to that of Ph + B-ALL^*IKZF1*+/*DDX41−*^ than to that of AML ^*DDX41dm*^, as shown in heatmap analysis (Fig. 3).

## Discussion and conclusions

Inherited *DDX41* mutations are always heterozygous and usually in frame-shift mutations, indicating a potential loss-of-function (LOF). Approximately half of MN patients with inherited *DDX41* mutations acquire a second-hit, often R525H, in the healthy *DDX41* allele in their disease clones [3]. To date, in all reported bi-allelic *DDX41* mutated HM cases, germline mutations rather than somatic mutations occurred relatively at the forefront of, except for an MDS case with germline R369G and somatic S4*[6]. In our case, the location of *DDX41* mutations differed from that in previous reports, where somatic and germline mutations occurred in the N-terminal domain and DEAD box domain, respectively.

Since *BCR-ABL1* translocation alone is insufficient for malignant transformation, it is known that various complex additional mutations are required for Ph + B-ALL development [7]. Over 70% of Ph + B-ALL patients harbor *IKZF1* LOF [7], however, to the best of our knowledge, concomitant *DDX41* mutations have never been reported. Furthermore, 5q deletion is also rarely observed in ALL, which we hypothesize to have resembled the GEP of our case, that deduction of *DDX41* mutation on lymphoid malignancy is challenging [8]. The previously reported cases have shown, DDX41 has been shown to be a cytoplasmic DNA sensor in dendritic cells and to have a documented role in the innate immune response [9]. Therefore, dysregulation of such responses may be an initiator of disorders and may be linked to lymphoid malignancy.

The interesting feature of our case was the concomitant mutation on *ABL1* (c.688C > T, p.Pro230Ser), which resides in the SH2-kinase linker domain of *ABL1* and is seldom observed in a *BCR-ABL1* transcript [10]. Association of *DDX41* mutations with this finding is uncertain,; however, *DDX41* mutations are largely mutually exclusive to with splice- factor mutations [11]. The loss of tumor suppressor function because of altered pre-mRNA splicing and RNA processing is another aspect of somatic *DDX41* mutations [2]. In our case, mutations were not detected in genes of the splice-factor family, and an explanation for this observation remains elusive.

We found altered gene expression levels of genes involved in the process essential for red blood cells, which is consistent with previous observations that *DDX41* mutations, including LOF, can affect erythroid differentiation [12]. For myeloid cell differentiation, Ph + B-ALL^*IKZF1*+/*DDX41dm*^ had significantly altered levels of expression compared to Ph + B-ALL^*IKZF1*+/*DDX41−*^. However, the contribution of *DDX41* mutations, including gain-of-function, to developing myeloid malignancy is not fully understood, but is presumed to be involved in the pathogenesis of a certain subset of such AML cases [13].

Based on case reports and retrospective analysis, lenalidomide has been suggested as an effective treatment strategy for myeloid malignancies with *DDX41* mutations [14]. The patient in our study has undergone a combination of modified CVAD and imatinib, which is front-line therapy for adult Ph + B-ALL [15]. Because of short follow-up time, whether the efficacy of lenalidomide might have been beneficial for this patient is could not be addressed. However, the genetic testing for *DDX41* to find an optimal family-member donor was performed done in a timely manner.

Herein, we report that *DDX41* mutations are unusual but can be an underlying event in Ph + B-ALL, although the causative link between *DDX41* variants and B-ALL is yet to be established; however, it shows heterogeneously transitional GEP of both Ph + B-ALL and AML with *DDX41* mutations. Screening *DDX41* mutations can be also informative for patients awaiting for haploidentical SCT and choosing the therapy.

## Supplementary Information

**Additional file 1:** The list of 85 hematologic malignancy associated genes included in customized NGS panel used this study

**Additional file 2:** Genetic alterations of the case series of this study

**Additional file 3:** A list of the 409 dysregulated transcripts for Ph + B-ALL^*IKZF1*+/*DDX41dm*^

**Additional file 4:** Description: Supplementary figure. (A) c.639delC, p.Thr214Profs*8 mutation of the patient's bone marrow sample (B) Same mutation found in the patient's skin fibroblasts (C) c.259C > T, p.Leu87Phe mutation of the patient’s bone marrow sample (D) The patient’s frameshift mutation confirmed by Sanger sequencing for bone marrow (E) Sanger sequencing for the sibling’s peripheral blood revealed presence of c.639delC.

## Data Availability

The datasets analyzed in the current study are available in the NCBI's Gene Expression Omnibus (GEO) with accession number GSE196107 (https://www.ncbi.nlm.nih.gov/geo/query/acc.cgi?acc=GSE196107) for gene expression and DDBJ Sequenced Read Archive (https://ddbj.nig.ac.jp/search), under the Bioproject accession number PRJDB13084 (Biosample: SAMD00443631- SAMD00443638) for genomic sequencing, respectively.

## Abbreviations

B-ALL: B lymphoblastic leukemia
GEP: Gene expression profiling
HM: Hematologic malignancy
BM: Bone marrow
WB: Whole blood
SCT: Stem cell transplantation
LOF: Loss-of-function
