# Supplementary material for: A novel bi-alleleic DDX41 mutations in B-cell lymphoblastic leukemia: case report
Source: BMC Med Genomics. 2022 Mar 4;15:46. doi: 10.1186/s12920-022-01191-2 (PMC8897883; doi:10.1186/s12920-022-01191-2)
Supplement: Supplementary file 1 — Additional file 1: The list of 85 hematologic malignancy associated genes included in customized NGS panel used this study [file 12920_2022_1191_MOESM1_ESM.doc]

**Additional data 1. The list of 85 hematologic malignancy associated genes included in customized NGS panel used this study**

*ABL1, ANKRD26, ASXL1, ATM, BCL2, BCL6, BCR, BIRC3, BRAF, CALR, CBFB, CBL, CCND1, CD58, CD79B, CDKN2A, CDKN2B, CEBPA, CREBBP, CSF3R, CTCF, DDX41, DIS3, DNMT3A, ETNK1, ETV6, EZH2, FGFR1, FLT3, GATA2, HRAS, ID3, IDH1, IDH2, IKZF1, JAK1, JAK2, JAK3, KANSL1, KIT, KMT2A, KMT2D, KRAS, LRP1B, LYL1, MAP2K1, MECOM, MEF2B, MPL, MYC, MYD88, NF1, NOTCH1, NOTCH2, NPM1, NRAS, NSD2, PAX5, PDGFRA, PDGFRB, PML, PRDM1, PTPN11, RARA, RB1, RPS19, RUNX1, SETBP1, SF3B1, SH2B3, SRSF2, STAT3, STAT5B, TAL1, TCF3, TENT5C, TET2, TLX1, TNFAIP3, TNFRSF14, TP53, TRAF3, U2AF1, WT1, XBP1*
