# Supplementary material for: A novel bi-alleleic DDX41 mutations in B-cell lymphoblastic leukemia: case report
Source: BMC Med Genomics. 2022 Mar 4;15:46. doi: 10.1186/s12920-022-01191-2 (PMC8897883; doi:10.1186/s12920-022-01191-2)
Supplement: Supplementary file 2 — Additional file 2: Genetic alterations of the case series of this study [file 12920_2022_1191_MOESM2_ESM.doc]

**Additional data 2. Genetic alterations of the case series of this study**

| Cases | Ph+B-ALL*^IKZF1^*^+/^*^DDX41^*^dm^ | Ph+B-ALL*^IKZF1^*^+/^*^DDX41-^* | Ph+B-ALL*^IKZF1^*^+/^*^DDX41-^* | AML*^DDX41dm^* |
| --- | --- | --- | --- | --- |
| Diagnosis | B lymphoblastic leukemia | B lymphoblastic leukemia | B lymphoblastic leukemia | Acute myeloid leukemia with minimal differentiation |
| Blast | 88.1 | 90.3 | 87.3 | 46.7 |
| Karyotype | 46,XY,t(9;22)(q34;q11.2)[10]/47,idem,+der(22)t(9;22)[2] | 47,XX,+5,t(6;18)(q23;q23),t(9;22)(q34;q11.2)[2]/48,idem,+der(22)t(9;22)[14]/48,idem,add(11)(q23),+der(22)t(9;22)[2]/48,idem,der(16)t(1;16)(q21;q22),+der(22)t(9;22)[2]/46,XX[1] | 48,XX,+5,del(9)(p22),t(9;22)(q34;q11.2),+der(22)t(9;22)[6] | 46,XY[20] |
| Immunophenotype | Positive for CD9, CD10, CD13, CD19, CD20, CD34, CD38, CD58, CD66c, CD123, HLA-DR, cCD79a, and TdT | Positive for CD10, CD13, CD19, CD20, CD38, HLADR, cCD79a, and TdT | Positive for CD9, CD10, CD19, CD20, CD34, CD38, CD58, CD66c, CD123, HLA-DR, cCD79a, and TdT | Positive for CD13, CD38, CD117, and HLA-DR. |
| Accompanied mutations and VAF (%) |  |  |  |  |
| Somatic | *DDX41* (c.259C>T, p.Leu87Phe) (39.7%) *IKZF1* exon 4-6 deletion (37.0%) *ABL1* (c.688C>T, p.Pro230Ser) (25.6%) | *RB1* exon 18-27 deletion (90.0%) *IKZF1* exon 4-8 deletion (45.0%) *IKZF1* c.40+2T>C, p.? (43.7%) | *IKZF1* exon 2-8 deletion | *DDX41* (c.1574G>A,p.Arg525His) (6.39) *SETBP1* (c.2602G>A,p.Asp868Asn) (8.81) *ASXL1* (c.2893C>T, p.Arg965*) (7.71) *CBL* (c.1201T>C, p.Cys401Arg) (7.03) |
| Germline | *DDX41* (c.639delC, p.Thr214Profs*8)  (40.0%) | - | - | *DDX41* (c.1496dupC, p.Ala500Cysfs*9) (6.39) |

VAF, variant allele frequency
