# Supplementary material for: A novel bi-alleleic DDX41 mutations in B-cell lymphoblastic leukemia: case report
Source: BMC Med Genomics. 2022 Mar 4;15:46. doi: 10.1186/s12920-022-01191-2 (PMC8897883; doi:10.1186/s12920-022-01191-2)
Supplement: Supplementary file 3 — Additional file 3: A list of the 409 dysregulated transcripts for Ph + B-ALLIKZF1+/DDX41dm [file 12920_2022_1191_MOESM3_ESM.doc]

***Additional data 3. A list of the 409 dysregulated transcripts for Ph+B-ALLIKZF1+/DDX41dm***

| Gene Symbol | mRNA Accession | Fold Change  (Ph+B-ALL*^IKZF1+/DDX41dm^* vs. Ph+B-ALL*^IKZF1+/DDX41-^*) | Gene Description | UniGene ID |
| --- | --- | --- | --- | --- |
| *PITHD1* | NM_020362 | 3.495490708 | PITH (C-terminal proteasome-interacting domain of thioredoxin-like) domain containing 1 | Hs.31819 |
| *RHD* | NM_001127691 | 6.935933181 | Rh blood group, D antigen | Hs.449968 |
| *EPB41* | NM_001166005 | 5.247553608 | erythrocyte membrane protein band 4.1 | Hs.175437 |
| *LINC01226* | NR_027085 | 10.20012552 | long intergenic non-protein coding RNA 1226 | Hs.658659 |
|  | ENST00000419428 | -3.311626572 |  |  |
| *FAM46C* | NM_017709 | 17.69506308 | family with sequence similarity 46, member C | Hs.356216 |
|  | ENST00000617273 | -3.749413655 |  |  |
| *RNVU1-3* | NR_104081 | -3.084250619 | RNA, variant U1 small nuclear 3 |  |
| *NBPF19* | XM_011508693 | -3.407845665 | neuroblastoma breakpoint family, member 19 |  |
| *NOTCH2NL* | ENST00000479995 | -3.525651804 | notch 2 N-terminal like | Hs.655156//Hs.728902 |
| *LOC101060254* | XM_011508668 | -5.075588119 | myomegalin-like |  |
| *RNVU1-3* | NR_104081 | -3.084250619 | RNA, variant U1 small nuclear 3 |  |
| *CD1C* | NM_001765 | 3.728434508 | CD1c molecule | Hs.132448 |
| *RALGPS2* | NM_001286247 | 3.216563121 | Ral GEF with PH domain and SH3 binding motif 2 | Hs.632485 |
|  | ENST00000414377 | -3.102680194 |  |  |
| *DTL* | NM_001286229 | 3.460702679 | denticleless E3 ubiquitin protein ligase homolog (Drosophila) | Hs.656473 |
| *FAM177B* | NM_207468 | -3.022191334 | family with sequence similarity 177, member B | Hs.697608 |
| *TRIM58* | NM_015431 | 10.0306278 | tripartite motif containing 58 | Hs.269151 |
| *OR2W3* | NM_001001957 | 3.015401271 | olfactory receptor, family 2, subfamily W, member 3 | Hs.269151 |
| *OR2L8* | NM_001001963 | 5.206666495 | olfactory receptor, family 2, subfamily L, member 8 (gene/pseudogene) | Hs.690212 |
| *OR2L5* | NM_001258284 | 6.706058182 | olfactory receptor, family 2, subfamily L, member 5 | Hs.742583 |
| *OR2M3* | BC136970 | 3.033934627 | olfactory receptor, family 2, subfamily M, member 3 | Hs.553581 |
|  |  | 4.164504929 |  |  |
| *UTS2* | NM_006786 | -8.812339032 | urotensin 2 | Hs.715862 |
|  | NONHSAT001567 | 3.864455089 |  |  |
|  | NONHSAT002276 | -3.598074354 |  |  |
|  | ENST00000459562 | -3.843937822 |  |  |
| *GBP2* | NM_004120 | -5.093421109 | guanylate binding protein 2, interferon-inducible | Hs.386567 |
| *GBP2* | NM_004120 | -3.285926021 | guanylate binding protein 2, interferon-inducible | Hs.386567 |
|  | ENST00000617273 | -3.749413655 |  |  |
| *NBPF13P* | OTTHUMT00000099375 | -9.075580972 | neuroblastoma breakpoint family, member 13, pseudogene |  |
| *SPTA1* | NM_003126 | 3.886226533 | spectrin, alpha, erythrocytic 1 | Hs.119825 |
|  | NONHSAT007749 | 3.058755944 |  |  |
| *ASPM* | NM_001206846 | 3.082124202 | abnormal spindle microtubule assembly | Hs.121028 |
| *MIR181A1HG* | NR_040073 | 3.015119119 | MIR181A1 host gene | Hs.711077 |
| *LINC01222* | NR_110525 | 9.686933247 | long intergenic non-protein coding RNA 1222 |  |
| *ADIPOR1* | NM_001290553 | 4.756053685 | adiponectin receptor 1 | Hs.713729 |
| *LYST* | NM_000081 | -4.782682431 | lysosomal trafficking regulator | Hs.532411 |
| *LYST* | NR_102436 | -5.199795913 | lysosomal trafficking regulator | Hs.532411 |
|  | NONHSAT010380 | -5.341648335 |  |  |
| *LINC01139* | NR_015407 | -6.370912398 | long intergenic non-protein coding RNA 1139 | Hs.532047 |
| *MCM10* | NM_018518 | 4.583008622 | minichromosome maintenance 10 replication initiation factor | Hs.198363 |
| *MAP3K8* | NM_001244134 | -4.102092431 | mitogen-activated protein kinase kinase kinase 8 | Hs.432453 |
| *MAP3K8* | XM_011519310 | -7.653369215 | mitogen-activated protein kinase kinase kinase 8 | Hs.432453 |
|  | ENST00000517245 | -4.079959977 |  |  |
| *NCOA4* | NM_001145260 | 4.223678432 | nuclear receptor coactivator 4 | Hs.643658//Hs.709644 |
| *SRGN* | NM_002727 | -3.343084915 | serglycin | Hs.1908 |
| *GRK5* | NM_005308 | -3.63264403 | G protein-coupled receptor kinase 5 | Hs.524625//Hs.736460 |
| *WDR11* | NM_018117 | -5.599449507 | WD repeat domain 11 | Hs.144447 |
| *LINC01163* | NR_120619 | 3.895733531 | long intergenic non-protein coding RNA 1163 |  |
|  | NONHSAT010849 | -4.925192114 |  |  |
|  |  | -3.261521186 |  |  |
| *PIP4K2A* | NM_005028 | 3.936886172 | phosphatidylinositol-5-phosphate 4-kinase, type II, alpha | Hs.57079 |
| *NRP1* | NM_001024628 | -5.831390757 | neuropilin 1 | Hs.131704 |
| *MARCH8* | NM_001002266 | 5.526534119 | membrane associated ring finger 8 | Hs.499489 |
|  | NONHSAT013186 | 6.697348308 |  |  |
|  | NONHSAT013939 | -3.152103704 |  |  |
| *WT1-AS* | NR_023920 | 3.372386759 | WT1 antisense RNA | Hs.567499 |
| *ALKBH3* | NM_139178 | -3.035217706 | alkB homolog 3, alpha-ketoglutarate-dependent dioxygenase | Hs.720708 |
| *ARHGEF12* | NM_001198665 | 3.183712364 | Rho guanine nucleotide exchange factor (GEF) 12 | Hs.24598 |
|  | ENST00000408104 | 8.063409549 |  |  |
| *HBD* | NM_000519 | 10.10120149 | hemoglobin, delta | Hs.699280 |
| *HBG1* | ENST00000330597 | 3.623791625 | hemoglobin, gamma A | Hs.702189 |
| *HBG2* | NM_000184 | 4.647033355 | hemoglobin, gamma G | Hs.302145 |
| *WT1* | NM_000378 | 3.500885797 | Wilms tumor 1 | Hs.591980 |
| *MS4A6A* | NM_001247999 | -4.647081671 | membrane-spanning 4-domains, subfamily A, member 6A | Hs.523702//Hs.744932 |
| *ARHGAP32* | NM_001142685 | -3.129907455 | Rho GTPase activating protein 32 | Hs.440379 |
|  | ENST00000422780 | 3.557774322 |  |  |
|  | ENST00000391130 | 5.140219616 |  |  |
| *EMP1* | NM_001423 | -3.649616818 | epithelial membrane protein 1 | Hs.719042 |
| *SLC35E3* | NM_018656 | 3.586284645 | solute carrier family 35, member E3 | Hs.506011 |
|  | NONHSAT030301 | -5.427320098 |  |  |
| *P2RX7* | NM_002562 | -3.003230069 | purinergic receptor P2X, ligand gated ion channel, 7 | Hs.729169 |
|  | NONHSAT027480 | -4.297030449 |  |  |
| *OVOS* | XM_011508343 | -4.143729994 | ovostatin | Hs.568152 |
| *ABCD2* | NM_005164 | 6.436337598 | ATP binding cassette subfamily D member 2 | Hs.117852 |
| *GTSF1* | NM_144594 | 9.090974807 | gametocyte specific factor 1 | Hs.524476 |
|  | ENST00000459514 | -12.23735618 |  |  |
| *ALDH1L2* | NM_001034173 | 3.412029216 | aldehyde dehydrogenase 1 family, member L2 | Hs.42572 |
|  | ENST00000364792 | -3.181815098 |  |  |
|  | ENST00000516319 | 3.242934759 |  |  |
| *SKA3* | NM_001166017 | 3.1108633 | spindle and kinetochore associated complex subunit 3 | Hs.88523 |
| *LINC00539* | NR_103840 | 3.547936035 | long intergenic non-protein coding RNA 539 | Hs.659198 |
| *RNU6-53P* | ENST00000365367 | -3.297436781 | RNA, U6 small nuclear 53, pseudogene |  |
| *TPT1* | NM_001286272 | 3.989602466 | tumor protein, translationally-controlled 1 | Hs.374596 |
| *POU4F1* | NM_006237 | 16.05621572 | POU class 4 homeobox 1 | Hs.654522 |
| *LIG4* | NM_001098268 | 5.184967694 | ligase IV, DNA, ATP-dependent | Hs.166091 |
| *TRDV2* | BC039714 | 3.896368157 | T cell receptor delta variable 2 | Hs.74647 |
| *TRAJ20* | OTTHUMT00000410978 | 4.784771402 | T cell receptor alpha joining 20 |  |
| *MMP14* | NM_004995 | -3.619586766 | matrix metallopeptidase 14 (membrane-inserted) | Hs.2399 |
| *ABHD12B* | NM_001206673 | 3.189211994 | abhydrolase domain containing 12B | Hs.271896 |
|  | ENST00000517241 | 6.594347892 |  |  |
| *VASH1* | NM_014909 | -3.128877118 | vasohibin 1 | Hs.525479 |
| *LINC00642* | NR_033986 | 4.549774365 | long intergenic non-protein coding RNA 642 | Hs.131037 |
|  | NONHSAT039234 | 6.700343227 |  |  |
| *TCL6* | NR_028288 | -3.198266149 | T-cell leukemia/lymphoma 6 (non-protein coding) | Hs.510368//Hs.732224 |
|  | NONHSAT036986 | 3.966358275 |  |  |
| *ADAM20P1* | NR_037933 | -3.287327062 | ADAM metallopeptidase domain 20 pseudogene 1 | Hs.188369 |
| *CDC42BPB* | NM_006035 | -3.518071978 | CDC42 binding protein kinase beta (DMPK-like) | Hs.654634 |
| *ZCWPW2* | AB087877 | -8.500160014 | zinc finger, CW type with PWWP domain 2 | Hs.539824//Hs.659030//Hs.510635 |
|  | AY062319 | 25.70104295 |  |  |
| *IGHV3-49* | OTTHUMT00000324613 | 13.93658323 | immunoglobulin heavy variable 3-49 |  |
| *SNORD116-21* | NR_003335 | -3.117360496 | small nucleolar RNA, C/D box 116-21 |  |
| *LOC100505534* | XR_109178 | 3.891523313 | uncharacterized LOC100505534 | Hs.636453 |
|  | NONHSAT041781 | -3.94215636 |  |  |
| *INAFM2* | NM_001301268 | 3.463030275 | InaF-motif containing 2 | Hs.530791 |
| *NEIL1* | NM_001256552 | 4.590129956 | nei-like DNA glycosylase 1 | Hs.512732 |
| *MCTP2* | NM_001159643 | 3.391162693 | multiple C2 domains, transmembrane 2 | Hs.33368 |
| *EPB42* | NM_000119 | 10.51447565 | erythrocyte membrane protein band 4.2 | Hs.368642 |
| *ZNF280D* | OTTHUMT00000419442 | 3.572490067 | zinc finger protein 280D | Hs.511477 |
|  | NONHSAT044125 | -5.126023171 |  |  |
| *HOMER2* | NM_004839 | -3.580782771 | homer scaffolding protein 2 | Hs.578443 |
| *HBM* | NM_001003938 | 4.397321452 | hemoglobin, mu | Hs.647389 |
| *HBA2* | NM_000517 | 3.697166459 | hemoglobin, alpha 2 | Hs.654744 |
| *HBA1* | NM_000558 | 4.945923756 | hemoglobin, alpha 1 | Hs.449630 |
| *AHSP* | NM_016633 | 39.96056289 | alpha hemoglobin stabilizing protein | Hs.274309 |
| *MT1F* | NM_001301272 | 3.569185787 | metallothionein 1F | Hs.513626 |
| *MT1H* | NM_005951 | 5.68773796 | metallothionein 1H | Hs.438462 |
| *CES3* | NM_001185176 | 3.054021037 | carboxylesterase 3 | Hs.268700 |
| *GABARAPL2* | NM_007285 | 3.719619377 | GABA(A) receptor-associated protein like 2 | Hs.461379 |
| *CENPN* | NM_001100624 | 3.388190519 | centromere protein N | Hs.726537 |
| *HAGH* | NM_001040427 | 3.004864633 | hydroxyacylglutathione hydrolase | Hs.157394 |
| *GINS2* | NM_016095 | 3.653236113 | GINS complex subunit 2 (Psf2 homolog) | Hs.433180 |
| *SLC7A5* | NM_003486 | 4.944809699 | solute carrier family 7 (amino acid transporter light chain, L system), member 5 | Hs.513797 |
|  | NONHSAT144537 | 3.0570179 |  |  |
| *GRAPL* | NM_001129778 | -6.406982278 | GRB2-related adaptor protein-like | Hs.661470//Hs.677674 |
|  | NONHSAT053937 | 5.024482456 |  |  |
| *MIR4729* | NR_039882 | -3.706326557 | microRNA 4729 |  |
| *MIR21* | NR_029493 | -4.29042332 | microRNA 21 |  |
| *TANC2* | NM_025185 | 3.004947947 | tetratricopeptide repeat, ankyrin repeat and coiled-coil containing 2 | Hs.410889 |
| *BAHCC1* | NM_001291324 | 5.995437359 | BAH domain and coiled-coil containing 1 | Hs.514580 |
|  | NONHSAT144845 | -5.084532039 |  |  |
| *SCIMP* | NM_001271842 | -33.84826545 | SLP adaptor and CSK interacting membrane protein | Hs.462080 |
| *SLFN14* | NM_001129820 | 8.863705819 | schlafen family member 14 | Hs.591193 |
| *SLC4A1* | NM_000342 | 30.36747393 | solute carrier family 4 (anion exchanger), member 1 (Diego blood group) | Hs.443948 |
| *SLC25A39* | NM_001143780 | 6.848651741 | solute carrier family 25, member 39 | Hs.744880 |
| *MXRA7* | NM_001008528 | -3.827705939 | matrix-remodelling associated 7 | Hs.250723//Hs.597019 |
|  | NONHSAT056070 | -6.62593936 |  |  |
|  | ENST00000610557 | -3.165558375 |  |  |
| *SNRPD1* | NM_001291916 | 3.578475246 | small nuclear ribonucleoprotein D1 polypeptide | Hs.464734 |
| *SETBP1* | NM_001130110 | -5.433229546 | SET binding protein 1 | Hs.435458 |
| *SLC14A1* | NM_001128588 | 7.617195902 | solute carrier family 14 (urea transporter), member 1 (Kidd blood group) | Hs.101307 |
| *PMAIP1* | NM_021127 | -3.033377393 | phorbol-12-myristate-13-acetate-induced protein 1 | Hs.96 |
|  | NONHSAT058883 | -3.300237841 |  |  |
|  | NONHSAT059123 | -4.984072312 |  |  |
| *FECH* | NM_000140 | 8.268267131 | ferrochelatase | Hs.365365 |
| *BSG* | NM_001728 | 4.82400031 | basigin (Ok blood group) | Hs.501293 |
| *OAZ1* | NM_001301020 | 4.716854176 | ornithine decarboxylase antizyme 1 | Hs.446427 |
| *UHRF1* | NM_001048201 | 3.150989616 | ubiquitin-like with PHD and ring finger domains 1 | Hs.108106 |
| *ASNA1* | NM_004317 | 3.433547685 | arsA arsenite transporter, ATP-binding, homolog 1 (bacterial) | Hs.465985 |
| *FAM129C* | NM_001098524 | 8.653657368 | family with sequence similarity 129, member C | Hs.434133 |
| *ZNF726* | NM_001244038 | 3.251634274 | zinc finger protein 726 | Hs.655305 |
| *GPI* | NM_000175 | -3.099122968 | glucose-6-phosphate isomerase | Hs.466471 |
| *FOSB* | NM_001114171 | -3.376210837 | FBJ murine osteosarcoma viral oncogene homolog B | Hs.590958 |
| *LILRA1* | NM_001278318 | -3.232577593 | leukocyte immunoglobulin-like receptor, subfamily A (with TM domain), member 1 | Hs.710507 |
| *SMIM24* | NM_001136503 | 3.449100101 | small integral membrane protein 24 | Hs.130714 |
| *KLF1* | NM_006563 | 3.759875784 | Kruppel-like factor 1 (erythroid) | Hs.37860 |
| *FKBP8* | NM_001308373 | 3.586036071 | FK506 binding protein 8 | Hs.173464 |
| *LINC01224* | NR_126448 | 4.046823702 | long intergenic non-protein coding RNA 1224 |  |
| *ZNF681* | NM_138286 | 3.726948801 | zinc finger protein 681 | Hs.399952//Hs.735840 |
|  | ENST00000383947 | -3.370236889 |  |  |
| *BLVRB* | NM_000713 | 13.8850455 | biliverdin reductase B | Hs.515785 |
| *MIR4324* | NR_036209 | -3.097158036 | microRNA 4324 |  |
| *SHANK1* | NM_016148 | 5.25434161 | SH3 and multiple ankyrin repeat domains 1 | Hs.274255 |
| *ZNF83* | NM_001105549 | -4.05233937 | zinc finger protein 83 | Hs.467210//Hs.665751//Hs.710125//Hs.744177 |
|  | ENST00000516525 | -4.310798117 |  |  |
|  | ENST00000620022 | 9.390845135 |  |  |
| *TRIB2* | NM_021643 | -4.5912914 | tribbles pseudokinase 2 | Hs.467751 |
| *MSGN1* | NM_001105569 | -3.658583029 | mesogenin 1 | Hs.705359 |
| *EPAS1* | NM_001430 | 3.780036067 | endothelial PAS domain protein 1 | Hs.468410 |
| *RPL23AP32* | NR_002229 | -8.336836259 | ribosomal protein L23a pseudogene 32 | Hs.657366//Hs.732136 |
|  | ENST00000363937 | -3.32211036 |  |  |
|  | ENST00000444852 | 5.001705104 |  |  |
|  | ENST00000459339 | 4.165197774 |  |  |
| *MAP4K4* | NM_001242559 | 3.305125377 | mitogen-activated protein kinase kinase kinase kinase 4 | Hs.701013 |
| *RNU4ATAC* | NR_023343 | -3.617191554 | RNA, U4atac small nuclear (U12-dependent splicing) | Hs.689638 |
|  | ENST00000432133 | -3.722701654 |  |  |
| *CDCA7* | NM_031942 | 5.542819394 | cell division cycle associated 7 | Hs.470654 |
|  | uc021vua.1 | -4.598011263 |  |  |
|  | uc021vul.1 | 3.575326512 |  |  |
| *SPATS2L* | NM_001100422 | -5.448673337 | spermatogenesis associated, serine-rich 2-like | Hs.120323 |
|  | NONHSAT076307 | -24.81249661 |  |  |
| *AOX2P* | NR_001557 | 3.518876789 | aldehyde oxidase 2 pseudogene | Hs.148269 |
| *STRADB* | NM_001206864 | 6.950202439 | STE20-related kinase adaptor beta | Hs.652338 |
| *SP140* | NM_001005176 | -4.715088991 | SP140 nuclear body protein | Hs.632549 |
| *AGAP1* | NM_001037131 | -3.632744749 | ArfGAP with GTPase domain, ankyrin repeat and PH domain 1 | Hs.435039 |
| *LOC102723927* | NR_110592 | 3.892359597 | uncharacterized LOC102723927 |  |
|  | NONHSAT068697 | -10.23138821 |  |  |
| *MBOAT2* | NM_138799 | 3.086164579 | membrane bound O-acyltransferase domain containing 2 | Hs.467634//Hs.593538 |
| *SMC6* | NM_001142286 | -3.214401183 | structural maintenance of chromosomes 6 | Hs.526728 |
| *LOC101929763* | XR_925574 | 22.80961198 | uncharacterized LOC101929763 |  |
|  | ENST00000421323 | 3.564858962 |  |  |
| *PELI1* | NM_020651 | -3.432750493 | pellino E3 ubiquitin protein ligase 1 | Hs.7886 |
| *SCN3A* | NM_001081676 | 4.247797524 | sodium channel, voltage gated, type III alpha subunit | Hs.435274 |
| *ALS2CR12* | NM_001127391 | 3.38567854 | amyotrophic lateral sclerosis 2 chromosome region candidate 12 | Hs.107944 |
| *TRAK2* | NM_015049 | 3.921593505 | trafficking protein, kinesin binding 2 | Hs.152774 |
|  | NONHSAT077257 | -7.857661395 |  |  |
| *RIN2* | NM_001242581 | -4.403375878 | Ras and Rab interactor 2 | Hs.472270 |
| *MYBL2* | NM_001278610 | 5.206666495 | v-myb avian myeloblastosis viral oncogene homolog-like 2 | Hs.179718 |
|  | ENST00000458830 | -3.561179115 |  |  |
| *FAM210B* | NM_080821 | 7.589027381 | family with sequence similarity 210, member B | Hs.143736 |
| *BCL2L1* | NM_001191 | 4.284004513 | BCL2-like 1 | Hs.516966 |
| *SAMHD1* | NM_015474 | -3.182542985 | SAM domain and HD domain 1 | Hs.580681 |
| *ADA* | NM_000022 | 3.275068314 | adenosine deaminase | Hs.654536 |
| *SULF2* | NM_001161841 | -4.736577533 | sulfatase 2 | Hs.162016 |
|  | ENST00000459405 | -3.258832045 |  |  |
| *ITGB2-AS1* | NR_038311 | -3.497575014 | ITGB2 antisense RNA 1 | Hs.661035 |
|  | NONHSAT081066 | -3.983771776 |  |  |
| *LOC101927745* | XR_920384 | 20.1053842 | uncharacterized LOC101927745 |  |
|  | ENST00000433588 | 4.767655521 |  |  |
| *TIAM1* | NM_003253 | -3.98036298 | T-cell lymphoma invasion and metastasis 1 | Hs.517228 |
| *LINC00114* | NR_027065 | 3.04087181 | long intergenic non-protein coding RNA 114 | Hs.278704 |
| *IL17RA* | NM_001289905 | -4.6241077 | interleukin 17 receptor A | Hs.48353 |
|  | ENST00000614584 | 6.857962426 |  |  |
| *BCRP3* | NR_024494 | 4.528882155 | breakpoint cluster region pseudogene 3 | Hs.655234 |
| *ADRBK2* | NM_005160 | 5.121122253 | adrenergic, beta, receptor kinase 2 | Hs.657494 |
| *SERHL2* | NM_001284334 | 3.814991916 | serine hydrolase-like 2 | Hs.728878 |
|  | ENST00000620909 | 3.578971363 |  |  |
|  | ENST00000417463 | 8.4060323 |  |  |
| *FGD5* | NM_152536 | -4.188199021 | FYVE, RhoGEF and PH domain containing 5 | Hs.412406 |
| *ARPP21* | NM_001025068 | 17.54739541 | cAMP-regulated phosphoprotein 21kDa | Hs.475902 |
|  | ENST00000364700 | -21.78419656 |  |  |
|  | ENST00000408132 | -6.590303927 |  |  |
|  | NONHSAT091187 | 3.570014663 |  |  |
| *CD86* | NM_001206924 | -3.154529845 | CD86 molecule | Hs.171182 |
| *MME* | NM_000902 | 3.383965828 | membrane metallo-endopeptidase | Hs.307734 |
|  | NONHSAT092876 | 5.324034795 |  |  |
|  | NONHSAT094172 | 4.546905436 |  |  |
| *LOC105376935* | XR_936563 | 4.49266036 | uncharacterized LOC105376935 |  |
|  | ENST00000390843 | -3.173918317 |  |  |
| *ROBO1* | NM_001145845 | -3.595207402 | roundabout guidance receptor 1 | Hs.744218 |
| *DPPA4* | NM_018189 | 3.172906482 | developmental pluripotency associated 4 | Hs.317659 |
| *GCSAM* | NM_001190259 | 3.444298072 | germinal center-associated, signaling and motility | Hs.49614 |
| *MYLK* | NM_053025 | 3.230438478 | myosin light chain kinase | Hs.477375 |
|  | NONHSAT091713 | 7.105174097 |  |  |
| *CEP70* | NM_001288964 | 3.108115249 | centrosomal protein 70kDa | Hs.531962 |
| *LOC105374150* | XR_924571 | 11.30704471 | uncharacterized LOC105374150 |  |
|  |  | 6.497163451 |  |  |
| *IGF2BP2* | NM_001007225 | 3.718781542 | insulin-like growth factor 2 mRNA binding protein 2 | Hs.35354 |
|  | ENST00000363314 | -8.11820066 |  |  |
|  |  | 3.160669093 |  |  |
| *ZNF141* | NM_003441 | -15.06031458 | zinc finger protein 141 | Hs.654355 |
|  | ENST00000362465 | -5.562351826 |  |  |
|  | NONHSAT095032 | -3.445694987 |  |  |
| *CD38* | NM_001775 | 3.394984529 | CD38 molecule | Hs.479214 |
| *SLIT2* | NM_001289135 | -4.38739622 | slit guidance ligand 2 | Hs.29802 |
| *MIR218-1* | NR_029631 | -5.313969694 | microRNA 218-1 |  |
|  | ENST00000410505 | -4.936351064 |  |  |
| *PRDM8* | NM_001099403 | -3.091967168 | PR domain containing 8 | Hs.373642 |
| *ARHGAP24* | NM_001025616 | 4.193456818 | Rho GTPase activating protein 24 | Hs.444229 |
| *BMPR1B* | NM_001203 | 14.9825886 | bone morphogenetic protein receptor type IB | Hs.598475 |
|  | AK058194 | 3.066366198 |  | Hs.531862 |
| *MND1* | NM_001253861 | 4.75550977 | meiotic nuclear divisions 1 | Hs.294088 |
| *SNX25* | NM_031953 | 6.629246966 | sorting nexin 25 | Hs.369091 |
| *PROM1* | NM_001145847 | -5.743857669 | prominin 1 | Hs.614734 |
| *TLR10* | NM_001017388 | -5.77381547 | toll-like receptor 10 | Hs.120551//Hs.730896 |
| *JCHAIN* | NM_144646 | 20.39827147 | joining chain of multimeric IgA and IgM | Hs.643431 |
| *PPBP* | NM_002704 | 3.999819786 | pro-platelet basic protein | Hs.2164 |
| *PIGY* | NM_001042616 | 3.133478295 | phosphatidylinositol glycan anchor biosynthesis class Y | Hs.26136 |
| *H2AFZ* | NM_002106 | 4.118103244 | H2A histone family, member Z | Hs.119192 |
|  | NONHSAT098101 | -3.067333427 |  |  |
| *TBC1D9* | NM_015130 | -4.475038207 | TBC1 domain family, member 9 (with GRAM domain) | Hs.480819//Hs.595450 |
|  | NONHSAT098510 | -20.83662916 |  |  |
| *GYPA* | NM_001308187 | 9.001466117 | glycophorin A (MNS blood group) | Hs.434973 |
| *NR3C2* | NM_000901 | -3.221270917 | nuclear receptor subfamily 3, group C, member 2 | Hs.163924 |
| *TMEM154* | NM_152680 | 3.908310261 | transmembrane protein 154 | Hs.518900 |
|  | NONHSAT099609 | -8.150790584 |  |  |
| *GAPT* | NM_001304428 | 3.087416245 | GRB2-binding adaptor protein, transmembrane | Hs.547697 |
| *CCNB1* | NM_031966 | 3.374210549 | cyclin B1 | Hs.23960 |
| *LOC728093* | XR_948446 | -3.049991031 | putative POM121-like protein 1-like | Hs.685195//Hs.729946//Hs.745184 |
|  | ENST00000408635 | -3.245835758 |  |  |
| *SMAD5* | NM_001001419 | -3.006041653 | SMAD family member 5 | Hs.167700 |
| *SNORA74A* | NR_002915 | -10.28988533 | small nucleolar RNA, H/ACA box 74A | Hs.684893 |
| *LOC105378210* | XR_944377 | 4.943832964 | uncharacterized LOC105378210 |  |
|  | NONHSAT104791 | -3.893614359 |  |  |
|  | ENST00000518103 | 4.023009096 |  |  |
| *LOC105377732* | XR_941239 | -30.69751569 | uncharacterized LOC105377732 |  |
| *LOC105377732* | XR_941238 | -3.003396608 | uncharacterized LOC105377732 |  |
| *LOC105377732* | XR_941238 | -7.373118786 | uncharacterized LOC105377732 |  |
| *RANBP3L* | NM_001161429 | -3.637532152 | RAN binding protein 3-like | Hs.729817 |
| *FYB* | NM_001243093 | 5.260937825 | FYN binding protein | Hs.370503 |
| *MCTP1* | NM_001002796 | -3.761987356 | multiple C2 domains, transmembrane 1 | Hs.655087 |
|  | ENST00000362916 | -4.07651126 |  |  |
| *CSF1R* | NM_001288705 | -3.634066954 | colony stimulating factor 1 receptor | Hs.586219 |
| *PDGFRB* | NM_002609 | -3.874486185 | platelet-derived growth factor receptor, beta polypeptide | Hs.509067 |
| *ZNF300* | NM_001172831 | 3.209970414 | zinc finger protein 300 | Hs.134885 |
| *ADAM19* | NM_033274 | -6.678735593 | ADAM metallopeptidase domain 19 | Hs.483944 |
| *HIST1H3A* | NM_003529 | 3.235491753 | histone cluster 1, H3a | Hs.546315 |
| *LOC102724851* | XR_427900 | -3.949554692 | uncharacterized LOC102724851 |  |
|  | NONHSAT108397 | -3.117641411 |  |  |
| *HIST1H2BM* | NM_003521 | 7.333363232 | histone cluster 1, H2bm | Hs.182432 |
| *HCG27* | ENST00000414008 | -3.524051481 | HLA complex group 27 (non-protein coding) | Hs.659818 |
| *COX6A1P2* | OTTHUMT00000040397 | 3.245284593 | cytochrome c oxidase subunit VIa polypeptide 1 pseudogene 2 |  |
| *TSPO2* | NM_001010873 | 4.178007262 | translocator protein 2 | Hs.357392 |
| *KCNQ5* | NM_001160130 | 3.726328854 | potassium channel, voltage gated KQT-like subfamily Q, member 5 | Hs.445324 |
| *KCNQ5-IT1* | NR_120503 | 3.498593383 | KCNQ5 intronic transcript 1 | Hs.662412 |
| *LOC105377927* | XR_942840 | 3.271540204 | uncharacterized LOC105377927 |  |
| *FAM26F* | NM_001010919 | 3.154180015 | family with sequence similarity 26, member F | Hs.381220 |
| *NRN1* | NM_001278710 | -3.004083679 | neuritin 1 | Hs.103291 |
| *HIST1H4D* | NM_003539 | 3.137640363 | histone cluster 1, H4d | Hs.248179 |
| *HIST1H3G* | NM_003534 | 5.524542513 | histone cluster 1, H3g | Hs.247813 |
| *HIST1H2BL* | NM_003519 | 3.398186422 | histone cluster 1, H2bl | Hs.137594 |
| *HIST1H2AJ* | NM_021066 | 3.464098613 | histone cluster 1, H2aj | Hs.406691 |
| *HIST1H1B* | NM_005322 | 3.673117379 | histone cluster 1, H1b | Hs.131956 |
| *HIST1H3I* | NM_003533 | 4.672371541 | histone cluster 1, H3i | Hs.132854 |
| *HIST1H2AM* | NM_003514 | 3.239227958 | histone cluster 1, H2am | Hs.134999 |
| *SUPT3H* | NM_001261823 | 4.836857383 | SPT3 homolog, SAGA and STAGA complex component | Hs.368325 |
|  | NONHSAT112977 | 8.970572123 |  |  |
| *RHAG* | NM_000324 | 3.068439206 | Rh-associated glycoprotein | Hs.120950 |
|  | NONHSAT113325 | -6.755113453 |  |  |
| *RRAGD* | NM_021244 | 3.216719193 | Ras-related GTP binding D | Hs.31712 |
|  | ENST00000384579 | 24.62778545 |  |  |
| *WASF1* | NM_001024934 | 3.079465574 | WAS protein family, member 1 | Hs.75850 |
| *IPCEF1* | NM_001130699 | -4.895597672 | interaction protein for cytohesin exchange factors 1 | Hs.146100 |
| *WDR27* | NM_001202550 | -4.476868679 | WD repeat domain 27 | Hs.131903 |
| *PHF10* | NM_018288 | -3.170839822 | PHD finger protein 10 | Hs.435933 |
|  | ENST00000420800 | -3.416146865 |  |  |
| *AHR* | NM_001621 | -3.459071896 | aryl hydrocarbon receptor | Hs.171189 |
|  | GENSCAN00000013425 | 6.192555957 |  |  |
| *LOC105375314* | XR_927568 | 3.326488393 | uncharacterized LOC105375314 |  |
| *LOC102724777* | XM_006716207 | 3.740432251 | putative uncharacterized protein FLJ92257 |  |
| *CDK14* | NM_001287135 | -3.513441799 | cyclin-dependent kinase 14 | Hs.258576 |
| *LOC101927902* | XR_242326 | -8.980277335 | uncharacterized LOC101927902 |  |
|  | NONHSAT122588 | -12.53373943 |  |  |
| *BPGM* | NM_001293085 | 4.115691939 | 2,3-bisphosphoglycerate mutase | Hs.198365 |
| *JHDM1D-AS1* | NR_024451 | 3.053872859 | JHDM1D antisense RNA 1 (head to head) | Hs.634333 |
| *TRBV20-1* | OTTHUMT00000352511 | 5.534526906 | T cell receptor beta variable 20-1 |  |
| *TRBJ2-6* | OTTHUMT00000352528 | -3.709269264 | T cell receptor beta joining 2-6 |  |
| *LOC105375113* | XR_926957 | -3.132859347 | uncharacterized LOC105375113 |  |
| *EIF2AK1* | NM_001134335 | 3.343351409 | eukaryotic translation initiation factor 2-alpha kinase 1 | Hs.520205 |
| *MEOX2* | NM_005924 | -5.029761527 | mesenchyme homeobox 2 | Hs.170355 |
| *LOC105375172* | XR_927075 | -4.226621726 | uncharacterized LOC105375172 |  |
| *TRGJP1* | OTTHUMT00000338835 | -7.476872855 | T cell receptor gamma joining P1 |  |
| *LOC100130849* | NR_038450 | 3.156968794 | phosphorylase kinase, gamma 1 (muscle) pseudogene | Hs.568145 |
| *NCF1C* | NR_003187 | -3.882161134 | neutrophil cytosolic factor 1C pseudogene | Hs.648940//Hs.655201 |
| *HGF* | NM_000601 | -3.076627967 | hepatocyte growth factor (hepapoietin A; scatter factor) | Hs.396530 |
| *PDK4* | NM_002612 | -8.484061036 | pyruvate dehydrogenase kinase, isozyme 4 | Hs.8364 |
| *RASA4B* | ENST00000488284 | 3.481683351 | RAS p21 protein activator 4B |  |
| *RASA4B* | XM_005250084 | 3.148588022 | RAS p21 protein activator 4B |  |
| *AASS* | NM_005763 | -4.485037352 | aminoadipate-semialdehyde synthase | Hs.156738 |
|  | NONHSAT123674 | 4.745318755 |  |  |
| *KEL* | NM_000420 | 4.863112614 | Kell blood group, metallo-endopeptidase | Hs.368588 |
| *MIR4659A* | NR_039803 | -3.06004952 | microRNA 4659a |  |
| *DMTN* | NM_001114135 | 5.619902106 | dematin actin binding protein | Hs.106124 |
| *SLC25A37* | NM_016612 | 4.166814861 | solute carrier family 25 (mitochondrial iron transporter), member 37 | Hs.596025//Hs.658208//Hs.726050 |
| *BNIP3L* | NM_004331 | 3.13705321 | BCL2/adenovirus E1B 19kDa interacting protein 3-like | Hs.131226 |
|  | ENST00000518535 | -3.75883347 |  |  |
|  | ENST00000518552 | -4.781290291 |  |  |
| *LOC105375903* | XR_929052 | 4.351476485 | uncharacterized LOC105375903 |  |
| *LOC102723716* | XR_428335 | -5.035168297 | uncharacterized LOC102723716 |  |
| *ANK1* | NM_000037 | 5.93700365 | ankyrin 1, erythrocytic | Hs.654438 |
| *TOX* | NM_014729 | -6.015563974 | thymocyte selection-associated high mobility group box | Hs.491805 |
|  | ENST00000364361 | -3.236635718 |  |  |
| *CA1* | NM_001128829 | 4.821209093 | carbonic anhydrase I | Hs.23118 |
| *LOC101929709* | NR_125822 | 4.031900399 | uncharacterized LOC101929709 |  |
|  | ENST00000522005 | -3.004864633 |  |  |
| *MTSS1* | NM_001282971 | -4.599190641 | metastasis suppressor 1 | Hs.336994 |
| *MIR3686* | NR_037457 | -3.784938871 | microRNA 3686 |  |
|  | ENST00000448570 | -4.115748995 |  |  |
| *NPR2* | XM_005251479 | -3.377404556 | natriuretic peptide receptor 2 | Hs.78518 |
| *LINC01507* | NR_121212 | 3.378341101 | long intergenic non-protein coding RNA 1507 |  |
| *TMOD1* | NM_001166116 | 6.236644392 | tropomodulin 1 | Hs.404289 |
| *ZNF462* | NM_021224 | 8.144296021 | zinc finger protein 462 | Hs.370379 |
| *BAG1* | NM_001172415 | 3.214066992 | BCL2-associated athanogene | Hs.377484 |
| *DCAF12* | NM_015397 | 10.35108433 | DDB1 and CUL4 associated factor 12 | Hs.493750//Hs.741270 |
| *MIR1299* | NR_031629 | -3.112610382 | microRNA 1299 |  |
|  | NONHSAT131838 | -3.413720638 |  |  |
| *HEMGN* | NM_018437 | 9.566624685 | hemogen | Hs.176626 |
| *ZNF883* | NM_001101338 | 3.677907 | zinc finger protein 883 | Hs.192877 |
| *DPM2* | NM_003863 | 3.08274381 | dolichyl-phosphate mannosyltransferase polypeptide 2, regulatory subunit | Hs.108973 |
| *LCN8* | NM_178469 | -6.539589145 | lipocalin 8 | Hs.323991 |
|  | ENST00000387392 | 7.617671102 |  |  |
|  | ENST00000387400 | 10.11475871 |  |  |
| *ND6* | ENST00000361681 | 8.896975626 | NADH dehydrogenase, subunit 6 (complex I) |  |
|  | ENST00000387459 | 3.048775669 |  |  |
|  |  | -5.651759203 |  |  |
|  | NR_003285 | -3.062500331 |  |  |
|  | NR_003285 | -3.062500331 |  |  |
|  | ENST00000410505 | -4.936351064 |  |  |
|  | ENST00000613295 | -3.749413655 |  |  |
| *XK* | NM_021083 | 4.188968397 | X-linked Kx blood group | Hs.78919 |
| *ZC3H12B* | XM_011530938 | 3.069673044 | zinc finger CCCH-type containing 12B | Hs.21249 |
| *H2BFXP* | uc010npa.1 | -7.602347649 | H2B histone family, member X, pseudogene | Hs.496530 |
| *IL13RA1* | NM_001560 | -8.441123312 | interleukin 13 receptor, alpha 1 | Hs.496646 |
|  | ENST00000458908 | -5.577582701 |  |  |
|  | ENST00000413328 | 3.06350881 |  |  |
| *SPANXB1* | NM_032461 | 3.098779284 | SPANX family, member B1 | Hs.711489 |
| *SPANXB1* | NM_032461 | 3.098779284 | SPANX family, member B1 | Hs.711489 |
| *SLC6A8* | NM_001142805 | 3.135618408 | solute carrier family 6 (neurotransmitter transporter), member 8 | Hs.540696 |
| *ALAS2* | NM_000032 | 43.34729312 | 5-aminolevulinate synthase 2 | Hs.522666 |
| *SLC7A3* | NM_001048164 | 16.38382153 | solute carrier family 7 (cationic amino acid transporter, y+ system), member 3 | Hs.175220 |
| *XIST* | NR_001564 | -315.1576779 | X inactive specific transcript (non-protein coding) | Hs.529901//Hs.655450 |
| *LOC286437* | NR_039980 | -4.766333829 | uncharacterized LOC286437 | Hs.656786 |
| *MBNL3* | NM_001170701 | 3.621468935 | muscleblind-like splicing regulator 3 | Hs.105134 |
| *CLIC2* | NM_001289 | 4.640852998 | chloride intracellular channel 2 | Hs.655445 |
| *XGY2* | NR_003254 | 3.25740928 | Xg pseudogene, Y-linked 2 | Hs.179675 |
| *TBL1Y* | NM_033284 | 12.57141379 | transducin (beta)-like 1, Y-linked | Hs.664560 |
|  | NONHSAT139356 | 3.116355891 |  |  |
| *TTTY15* | NR_001545 | 8.800435994 | testis-specific transcript, Y-linked 15 (non-protein coding) | Hs.433656 |
| *USP9Y* | NM_004654 | 6.419762831 | ubiquitin specific peptidase 9, Y-linked | Hs.598540 |
| *DDX3Y* | NM_001122665 | 14.25958411 | DEAD (Asp-Glu-Ala-Asp) box helicase 3, Y-linked | Hs.99120 |
| *TXLNGY* | NR_045128 | 9.46734568 | taxilin gamma pseudogene, Y-linked | Hs.522863 |
| *EIF1AY* | NM_001278612 | 3.590077529 | eukaryotic translation initiation factor 1A, Y-linked | Hs.461178 |
| *UTY* | NM_001258249 | 10.50052823 | ubiquitously transcribed tetratricopeptide repeat containing, Y-linked | Hs.115277 |
| *KDM5D* | NM_001146705 | 8.020376708 | lysine (K)-specific demethylase 5D | Hs.80358 |
|  | ENST00000624109 | 5.517443687 |  |  |
| *LOC100130428* | AY358681 | -3.598685434 | IGYY565 | Hs.661469 |
| *LOC285097* | XR_108456 | 4.168634835 | uncharacterized FLJ38379 | Hs.653239 |
| *TXLNGY* | NR_045128 | 8.474715869 | taxilin gamma pseudogene, Y-linked | Hs.522863 |
