# Supplementary figures and images for: A novel bi-alleleic DDX41 mutations in B-cell lymphoblastic leukemia: case report
Source: BMC Med Genomics. 2022 Mar 4;15:46. doi: 10.1186/s12920-022-01191-2 (PMC8897883; doi:10.1186/s12920-022-01191-2)

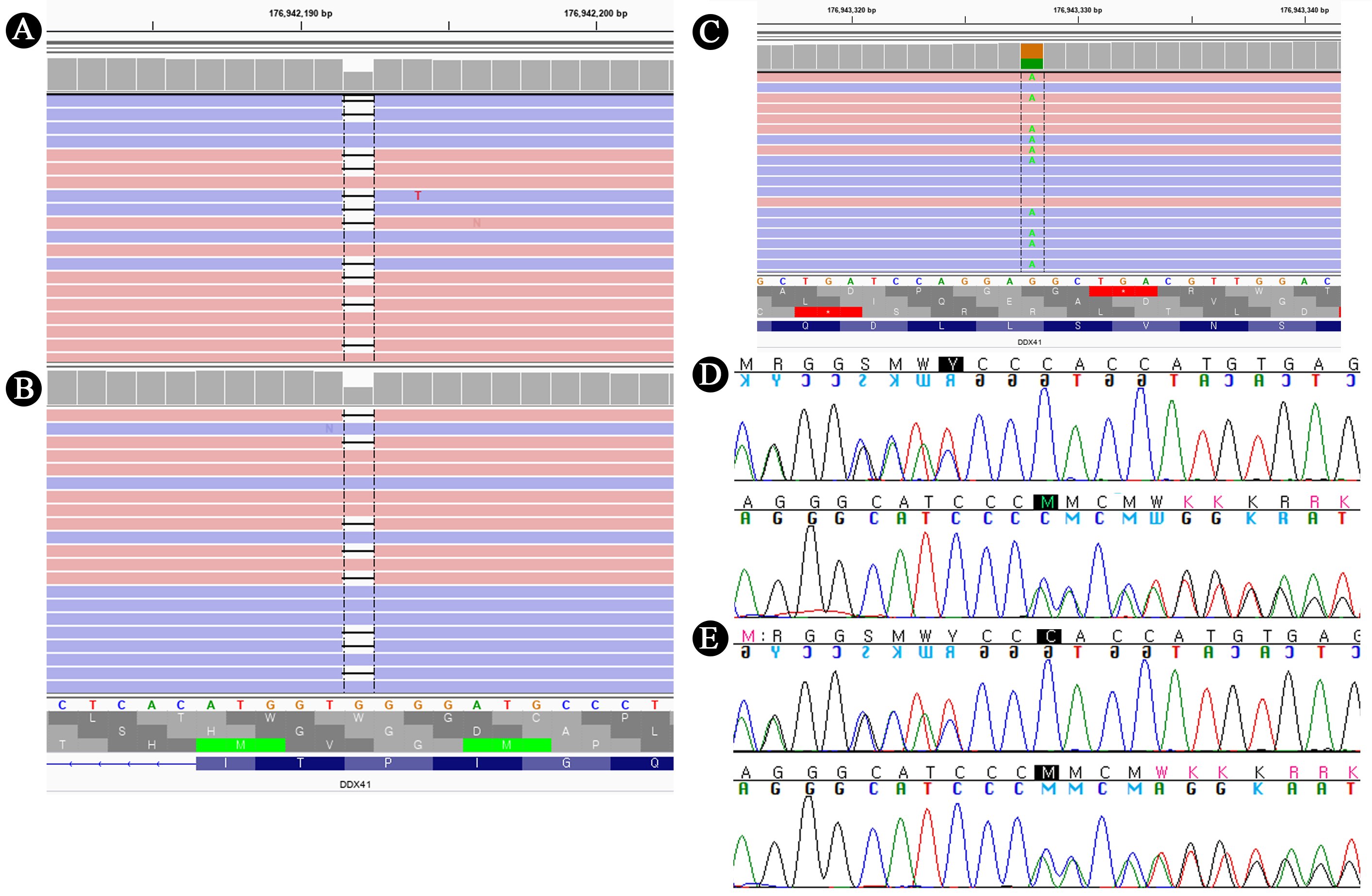

Supplement: Supplementary file 4 — Additional file 4: Description: Supplementary figure. (A) c.639delC, p.Thr214Profs*8 mutation of the patient's bone marrow sample (B) Same mutation found in the patient's skin fibroblasts (C) c.259C > T, p.Leu87Phe mutation of the patient’s bone marrow sample (D) The patient’s frameshift mutation confirmed by Sanger sequencing for bone marrow (E) Sanger sequencing for the sibling’s peripheral blood revealed presence of c.639delC. [file 12920_2022_1191_MOESM4_ESM.png]
